# Effect of Mass Reduction of 3D-Printed PLA on Load Transfer Capacity—A Circular Economy Perspective

**DOI:** 10.3390/ma18143262

**Published:** 2025-07-10

**Authors:** Aneta Liber-Kneć, Sylwia Łagan

**Affiliations:** 1 Faculty of Mechanical Engineering, Department of Applied Mechanics and Biomechanics, Cracow University of Technology, al. Jana Pawła II 37, 31-864 Kraków, Poland; aneta.liber@pk.edu.pl; 2 Interdisciplinary Center for Circular Economy, Cracow University of Technology, ul. Warszawska 24, 31-155 Kraków, Poland

**Keywords:** polylactide, 3D printing, mechanical properties, digital image correlation, stress relaxation

## Abstract

(1) Background: Optimizing infill density in 3D-printed PLA parts reduces material usage, cost, and waste. This study examines mechanical behavior in the initial and hydration stages. The findings provide valuable data for numerical simulations and engineering applications in additive manufacturing. (2) Methods: PLA specimens were printed with infill densities of 100%, 75%, and 25%. Mechanical tests, including tensile and compression tests, and one-hour stress-relaxation at 2% strain were conducted. The digital image correlation method was used to obtain the strain fields on the samples’ surface under tensile loading. Mechanical properties, including the elastic modulus, strength values, and Poisson’s ratio, were assessed. Hydrolytic degradation effects over one month were also evaluated. (3) Results: Lowering the PLA infill density reduced the ultimate tensile strength (from 60.04 ± 2.24 MPa to 26.24 ± 0.77 MPa), Young’s modulus (from 2645.05 ± 204.15 MPa to 1245.41 ± 83.79 MPa), compressive strength (from 26.59 ± 0.80 MPa to 21.83 ± 1.01 MPa), and Poisson’s ratio (from 0.32 to 0.30). A 40% mass reduction (form 100% to 25% infill density) resulted in a 56% decrease in tensile strength and a 53% decrease in Young’s modulus. A 31% mass reduction was observed for compression samples. Stress relaxation decreased significantly from 100% to 75% density, with further reductions having minimal impact. Hydrated samples showed no mechanical changes compared to baseline specimens. (4) Conclusions: Optimizing infill density in 3D-printed PLA parts helps to balance mechanical performance with material efficiency. The best mechanical properties are typically achieved with an infill density of 100%, but results show that decreasing the mass of the part by a reduction in infill density from 75% to 25% does not significantly affect the ability to transfer tensile and compression loads. PLA’s biodegradability makes it a viable alternative to stable polymers. By minimizing material waste and enabling the efficient use of resources, additive manufacturing aligns with the principles of a closed-loop economy, supporting sustainable development.

## 1. Introduction

Plastics production exceeded the level of 400 million tons in 2023, and the trend of yearly production increases observed since 1950 suggests further growth [1]. Increasing production of plastic goods has led to the generation of around 350 million tons of plastic waste each year [2]. This shows the critical need to implement a circular plastic economy. The circular plastic economy promotes the flow of plastics in a closed cycle, in which plastic goods at the end of life will turn into resources, minimizing plastic pollution. A circular plastic economy can fall into several models: (1) reuse and extend service life through repair or remanufacture; (2) recycling—turning old goods into new resources; (3) reducing the use of plastic or replacing petroleum-based materials with biobased and biodegradable polymers [3,4].

Substituting plastics derived from fossil resources with polymers from renewable resources brings several benefits related to the reduction of greenhouse gas emissions and obtaining polymers that are easier to recycle at the end of their life through the process of biodegradation [5]. The most commonly produced bioplastics are polylactic acid (PLA), polyhydroxyalkanoates (PHAs), starch, cellulose, and protein-based polymers [6]. Such polymers can be recycled through a degradation process in water, soil, and industrial composting systems, especially in cases where plastics become highly contaminated and are difficult to recover. Industrial composting systems meet the ISO 17088 [7] requirements for degradation (temperature, humidity, and microbial blends), the percentage of CO_2_ emitted from bioplastics, and any toxic residues remaining. Among biodegradable polymers, polylactic acid draws the most attention. PLA is a biodegradable and biocompatible thermoplastic polyester, derived from renewable resources such as cornstarch and sugarcane. The use of natural raw materials means that PLA can degrade to carbon dioxide and water or lactide acid oligomers, reducing the environmental impact associated with plastic waste. The production of PLA through fermentation process in controlled polymerization of lactic acid monomers decrease carbon emission. Thus, it has become a promising alternative to conventional oil-based plastics [8,9]. In the context of a circular economy, PLA can be considered as a special polymer, because it can be treated at all levels of the waste management hierarchy including source reduction (reuse), recycling (mechanical or chemical), incineration with energy recovery, and composting at the end of its life [10,11,12].

The increased interest in PLA is due to its competitive prices, ease of processing, and biodegradability. These features also contribute to its industrial applications, which can be divided into two groups for economic reasons based on its useful life: long-lasting (useful life > 3 years) and short-lasting (useful life < 3 years) [10]. The PLA’s properties and performance make it suitable for food packaging application, and in this aspect, it is widely used (except for carbonate beverage bottles) [13]. Another application area for PLA is the automotive sector, with the majority of PLA applications in automotive components, including items such as front panels, floor mats, car seats, pillar covers, door trims, and headliners [14]. Another field of application for biodegradable polymers, including PLA, is plasticulture, where the waste handling of conventional oil-based plastics carries risks of pesticide contamination and can limit their recycling. Thus, the use of biodegradable materials in this area is becoming increasingly popular [10]. PLA is also widely used in many different areas of medicine [10,15,16], such as orthopedics [17], dentistry [18,19], and ophthalmology [20]. Depending on its intended use, it can act as a temporary implant to support the healing process, e.g., as a cellular scaffold for bone reconstruction [21], as an attachment for ruptured ligaments or tendons [22], or in vascular stents [23], and, most importantly, in surgical sutures [24], which can biodegrade and be bioresorbable as the body takes over their function. The popularity of PLA in medicine is related not only to its biodegradability (decomposed into CO_2_ and H_2_O) and versatility of application but also to its biocompatibility (compatible with living tissues) and good strength properties [15]. PLA is also used in prosthetics as an alternative to the traditional composite socket in femoral stem prosthesis [25,26], and in upper limb orthoses and prostheses [27].

Despite the great promise of using PLA, there are still many challenges related to its unsatisfactory properties (e.g., mechanical, thermal) for given applications [28,29] and the design of cost-effective processing into intended forms and structures [10,30].

An equally important circular economy issue is the process of converting PLA into finished products. One of the methods of processing PLA is fused deposition modeling (FDM), an additive technology that can be used to create complex structures with great precision. A circular plastic economy also addresses additive manufacturing as a technology used in biodegradable polymer processing [4] and can be an option for polymer recycling processes by using 3D-printing filaments derived from plastic waste [31,32]. The 3D printing of PLA is associated with many parameters that should be optimized to obtain a good performance of produced parts. Many studies show the influence of 3D-printing parameters on the mechanical behavior of parts made from PLA [4,33,34]. Authors have demonstrated that the mechanical performance of printed samples is influenced by infill density, infill pattern, raster angle, layer thickness, nozzle temperature, and bed temperature.

The results of the investigation conducted on 3D-printed PLA by Rodríguez-Panes et al. [33] showed that increasing the infill density improves mechanical strength, but a larger layer height had the opposite effect. A higher layer thickness reduces the strength of the component but, on the other hand, results in a shorter printing time. Orienting the layers perpendicular to the load direction had a negative effect on the tensile strength. Among the three-dimensional printing parameters analyzed, the authors identified filling density (the quantity of material with which the component is 3D-printed) as the key parameter. Albadrani compared the mechanical properties of 3D-printed PLA with 15° and 30° raster angles and reported that by selecting raster angles in the desired directions for printed objects, it is possible to enhance their strength and stiffness [35]. Alhazmi et al. [34] analyzed PLA printed dog bone specimens with three different raster orientations (±45°, 45°, and 0°) and infill densities (20%, 40%, 60%, 80%, and 100%). The optimal mechanical performance for PLA was achieved with a raster orientation of ±45°, showing higher values of tensile strength and Young’s modulus in comparison to other orientations. Considerable variation in Young’s modulus and tensile strength was observed at 40%, 60%, and 80% infill densities, showing the crucial role of this parameter. Kuclourya et al. [4] estimated that the tensile strength of 3D-printed PLA had a direct relationship with infill density. When moving from 30% infill to 60% infill, the tensile strength increased by 15%. It was also observed that at a 0°/90° raster angle orientation, the tensile strength was the highest, with a decrease at a ±45° raster angle.

Knowing the load capacity that 3D-printed PLA parts can transfer leads to a reduction of the infill density, and thus the amount of material used. This approach has several advantages during production, such as less material use and reduced manufacturing time. These factors influence cost reduction and decrease end-of-life waste. Therefore, in order to achieve a low weight and high strength of the product, it becomes necessary to obtain an optimal infill density. Recent studies on lightweight design in fused deposition modeling (FDM) have highlighted diverse strategies to reduce part mass without compromising structural integrity. Researchers have explored the role of variable infill densities in optimizing mechanical performance, while advances in biopolymer materials have introduced new sustainability dimensions to lightweight fabrication [4,33,34,36]. Additionally, topology optimization algorithms have become instrumental in distributing material more efficiently within printed components.

However, despite these developments, the literature insufficiently addresses the influence of mass reduction on load transfer behavior. The article concentrates on the influence of infill density on the mechanical properties of PLA printed specimens under tensile and compression load to indicate the optimal relationship between the mass of printed parts and the capacity for load transfer. This gap is critical, as changes in weight distribution can significantly alter how loads are transmitted through a part, potentially leading to unanticipated stress concentrations or failure modes. Further investigation is necessary to understand this relationship and ensure reliable design in weight-sensitive applications. An additional aspect was to carry out stress-relaxation tests, as well as to determine the Poisson’s ratio using the digital image correlation method. This comprehensive approach to assessing the mechanical properties for different stress states provides data that can be useful for numerical analyses under different load states. The effect of hydration on mechanical properties was initially assessed by testing samples exposed to the liquid for one month. In our work, we analyze tensile and compressive loads for the samples with the same printing parameters, whereas other works focus on a single load scheme, which makes comparison of the results difficult.

## 2. Materials and Methods

### 2.1. Materials

The test specimens (for compression and tensile tests) were made using the FDM (fused deposition modeling) method. The 3D printer MarketBot SKETCH (MarketBot Industries, LLC, New York, NY, USA) was used with a working area of 150 × 150 × 150 mm and a layer resolution of 100–400 μm with UltiMaker Cura Cloud software V5.2 (UltiMaker, Zaltbommel, The Netherlands). The PLA Starter filament (ROSA PLAST SP. z o.o., Hipolitow, Poland), odorless, with a white color, a diameter of 1.75 mm, a density of 1.24 g/cm^3^, and a heat distortion temperature of 55 °C, was used. The printing parameters are presented in Table 1.

**Figure 1:**
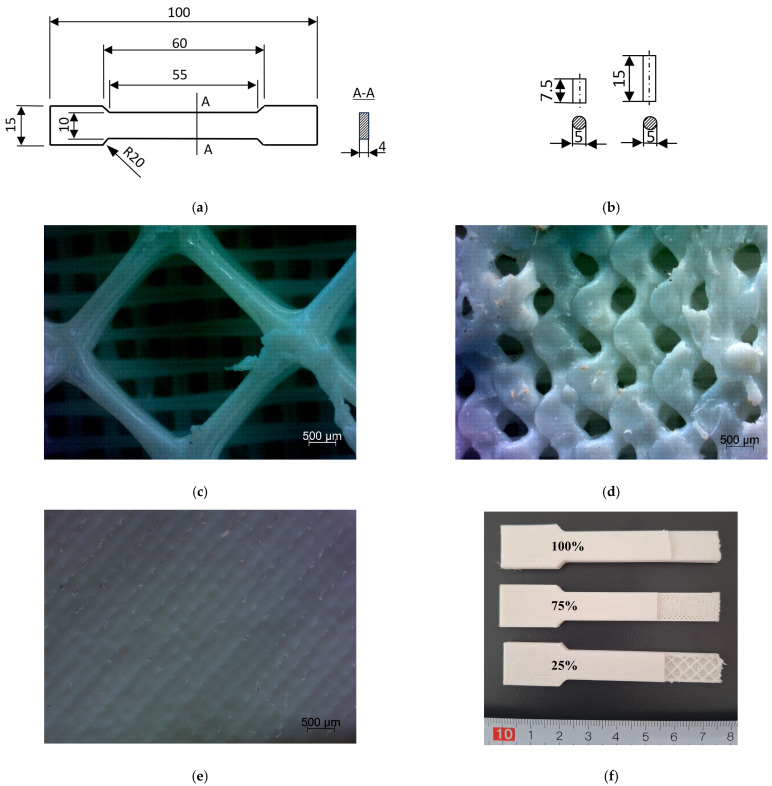
Printed specimens: (**a**) for tensile test, (**b**) for compression test, (**c**) internal structure of the sample with an infill density of 25%, (**d**) internal structure of the sample with an infill density of 75%, (**e**) internal structure of the sample with an infill density of 100%, (**f**) actual printed samples without outer layer.

The Diamond Fill Fast pattern is a type of lattice with a geometric arrangement of diamonds. It features a repetitive layout of rhombuses or diamonds, which can be filled with various textures or left open, creating a mesh-like effect. Two types of specimens with infill densities of 100%, 75%, and 25% (Figure 1) were printed: dumbbell-shaped samples (Figure 1a) for tensile tests and two types of cylindrical specimens differing in their height-to-diameter ratio, i.e., 1.5× and 3× (Figure 1b) for compression tests. Tensile test samples were made in accordance with ISO 527-2 [37] with regard to cross-sectional dimensions and gauge length, and the width and length of the grip section of the specimen were reduced. The samples were printed in a single build orientation, known as the flat printing orientation, relative to the plane of the printer table in the Cartesian reference system, where XY determines the plane of the table and Z direction is perpendicular to the printer table. Each printed sample had two bottom/top layers with a thickness of 0.72 mm for outer layer and 0.36 mm for invisible layer. Every layer of infill pattern had the thickness of 0.2 mm.

### 2.2. Methods

#### 2.2.1. Mechanical Tests

An MTS Insight 50 (MTS Insight^TM^, Eden Prairie, MN, USA) universal testing machine with a ±50 kN load cell and the Test Works 4.0 software was used for tensile (in accordance with ISO 527 standard [37]) and compression tests. The compression speed was 0.5 mm/min. The specimens (n = 5) were compressed until their height was shortened by 2.0 mm. The tensile test speed was 5 mm/min and the gauge length was 60 mm. Samples (n = 5) were subjected to tension until destruction. One-hour stress-relaxation tests were also performed at 2% of tensile strain for PLA samples with three different infill densities (100%, 75%, and 25%). All tests (compression, tensile, and stress relaxation) were carried out at a room temperature of 20 ± 1 °C and 40% humidity. After mechanical tests, the values of ultimate tensile strength (UTS) and compressive strength (σ_c_) were determined and the values of elastic modulus (Young’s modulus (E) and compressive modulus (E_c_)). The tensile strength-to-weight ratio, and the tensile stiffness-to-weight ratio (both in MPa/g), of each specimen was determined by dividing the peak tensile stress (UTS*), and Young’s modulus (E*) by the specimen mass, m (g), respectively. The value of residual stress (in MPa) after 1 h relaxation was obtained in initial and after incubation stage, and the normalized stress (-) curve were calculated by dividing the stress-relaxation data point by the peak tensile stress corresponding to 2% of stretching and mass of each specimen.

In addition, the effect of one-month hydrolytic degradation on the mechanical properties was considered.

#### 2.2.2. Digital Image Correlation (DIC)

**Figure 2:**
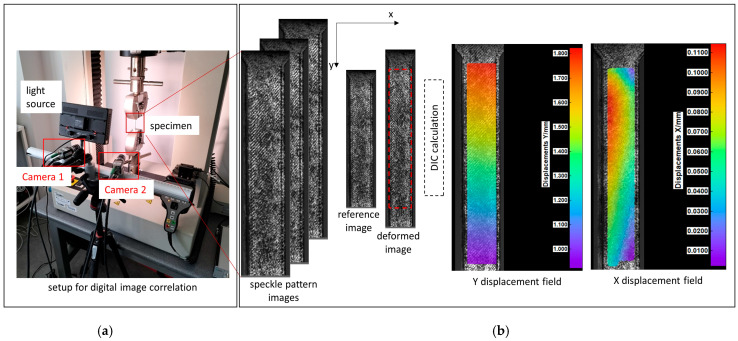
Two-dimensional digital image correlation: (**a**) system configuration, (**b**) in-plane displacement field calculation.

In order to assess the deformation capacity of the specimens across the entire surface during stretching and obtain values of Poisson’s ratio, the method of digital image correlation (DIC) was used (Figure 2). The DIC method is an optical, non-contact measurement tool used to assess displacement and strain in three-dimensional space. The DIC system (Dantec Dynamics GmbH, Ulm, Germany) consisted of two 2 MPx cameras and the DIC data processing system Istra4D V4.8.2.248 (Dantec Dynamics GmbH, Ulm, Germany). All samples were covered with a speckle pattern (base surface—matte white acrylic paint, dots—spraying matte black acrylic paint). Calibration was performed before the tests using a proprietary calibration target (Al11-BMB-9 × 9, Dantec Dynamics GmbH, Ulm, Germany). The size of the camera’s field of view was limited to the region of interest (ROI) (about 80% of sample surface) to avoid edge effects. During the tensile test, the cameras tracked the movement of the speckle pattern and then during the analysis, the recorded images were transformed into a 3D surface mesh using stereo-triangulation. By correlating the reference surface mesh (for the unloaded specimen) and the subsequent generated meshes, a map of the displacement and deformation on the specimen surface in three directions was obtained. The Poisson’s ratio was calculated and the base of strain values for longitudinal and transversal directions to force direction.

#### 2.2.3. Hydration Test

From the group of specimens of each infill density intended for tensile and compression tests (15 mm height), five specimens were placed in Ringer’s solution (Serumwerk Bernburg AG, Benburg, Germany): sodium chloride 8.6 g, potassium chloride 0.3 g, calcium chloride dihydrate 0.33 g 96 (^mmol/L: Na^+^ 147.2, K^+^ 4, Ca^++^ 2.25, Cl^−^ 155.7), pH value 5.0–7.5, acidity (titration to pH 7.4) <0.1 mmol/L, theoretical osmolarity 307 mOsm/L at 37.0 ± 0.1 °C in a thermal chamber (AL01-02-100 Advantage Lab, Schilde, Belgium) for 30 days. The volume of the solution was 100 mL for the compression tests specimens (cylindrical shape) and 500 mL for the tensile tests specimens (dumbbell-shaped). The mass of the samples was controlled using an analytical balance (AS 160/C/2, Radwag, Radom, Poland) with an accuracy of 1 × 10^−4^ g, before and after the incubation period. The degree of mass change was assessed as the quotient of the post-incubation mass change (the difference between the initial and post-incubation mass) and the initial weight. The initial mass of the dumbbell-shaped specimens was 4.2979 ± 0.7499 g, 3.9889 ± 0.6294 g, and 3.6786 ± 0.0862 g, and that of the 15 mm high cylindrical specimens was 0.2339 ± 0.042 g, 0.2087 ± 0.0023 g, and 1.685 ± 0.0059 g for infill densities of 100%, 75%, and 25%, respectively. After the 30-day incubation process, the degree of absorption was determined.

#### 2.2.4. Statistical Analysis

The data presented in this study were the mean values ± standard deviation determined by testing five samples for each case considered. Significant statistical differences between groups of data were determined using Student’s *t*-test. The level of significance used was 95 per cent (i.e., *p* ≤ 0.05). The basic assumptions of Student’s *t*-test were checked, i.e., equality of groups (same number of observations) and homogeneity of variance (Fisher’s test).

## 3. Results

**Figure 3:**
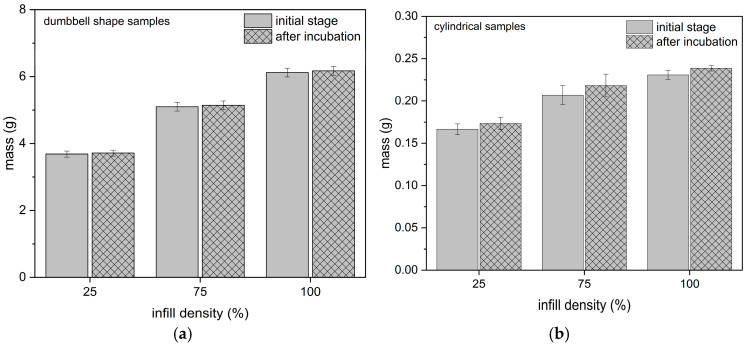
The mass change for (**a**) dumbbell-shaped samples, (**b**) cylindrical samples (3× ratio).

The change in mass of dumbbell-shaped samples and cylindrical samples after 30 days of hydration is shown in Figure 3. Evaluation of the effect of the short-term hydration of the samples showed that, regardless of the level of filling, the samples showed stability and their weight changes were not significant. This was confirmed in the mechanical properties tests, where the tested parameters for hydrated samples did not change compared to the base samples.

The use of different levels of infill density resulted in a mass redaction of 40% between samples with a 100% and 25% infill density (for samples used for tensile and relaxation tests), and for cylindrical samples a reduction of 28% of the mass for triplicate samples and 33% for 1.5× samples.

### 3.1. Tensile and Compression Tests

**Figure 4:**
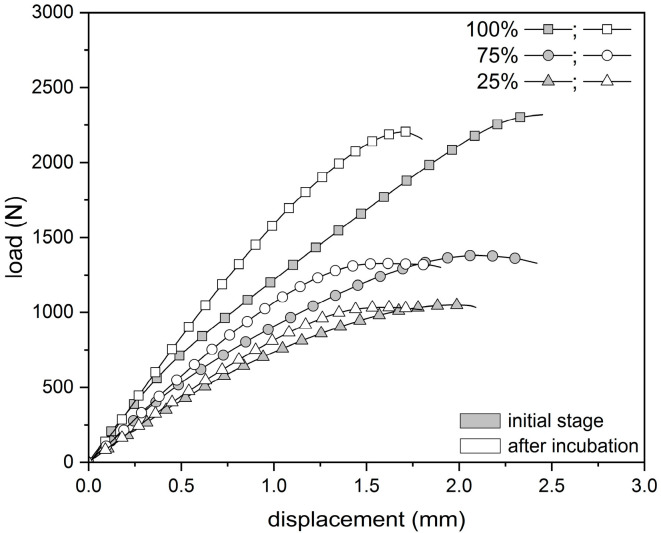
Average tensile curves for PLA specimens.

During the tensile test, all samples (regardless of infill density) were characterized by a nonlinear response of the material to loading (Figure 4). The highest values of the breaking force were obtained for samples in their initial state with 100% filling (2401.4 ± 89.7 N). Samples with a 75% infill of density obtained 1386.3 ± 63.7 N, while the group with the lowest infilling density obtained 1049.8 ± 30.9 N. The recorded extension (corresponding to the maximum force) was at the level of 2.5 ± 0.2 mm, 2.2 ± 0.2 mm, and 2.0 ± 0.1 mm for samples with a 100%, 75%, and 25% density of infill, respectively. In each group (100%, 75%, and 25%), the 30-day incubation caused a decrease in the value of the maximum force by 8%, 5%, and 1%, respectively, and in the corresponding elongation by 32%, 27%, and 20%, respectively. The infill density of the specimen had a significant effect on the tensile curve, whereas the effect of hydration was only significant for specimens with a 100% infill.

**Figure 5:**
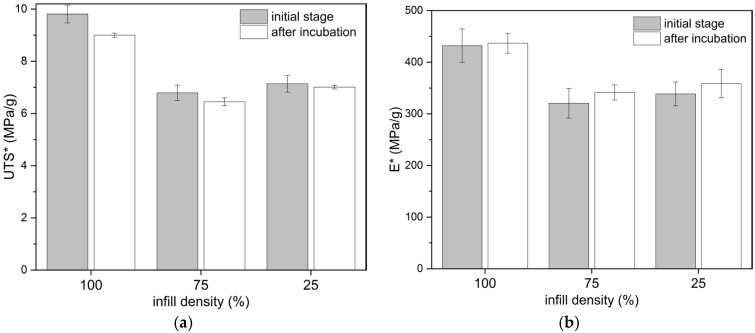
Comparison of the influence of infill density of PLA dumbbell-shaped specimens on the tensile properties in relation to the mass: (**a**) ultimate tensile strength (UTS*), (**b**) Young’s modulus (E*).

The tensile tests of PLA samples with different infill densities showed a decrease in tensile strength and modulus of elasticity values along with a decrease in infill density (Table 2). In relation to a 100% infill density, the UTS values decreased by 42.27% (PLA_75) and 56.29% (PLA_25) for a corresponding mass reduction of 7.18% and 14.41%, respectively. The effect of hydration on tensile strength was statistically significant only for a 100% infill density. As shown in Figure 5, the tensile-strength-to-weight ratio (UTS*) and the tensile stiffness-to-weight ratio (E*) relating the obtained parameters to the mass of the samples resulted in a significant decrease in their values when the filling density was reduced to 75%. A further reduction in infill density from 75% to 25% did not significantly reduce the analyzed values. The values of specific strength were 9.8 ± 0.3 MPa/g, 6.8 ± 0.3 MPa/g, and 7.4 ± 0.3 MPa/g for the 100%, 75%, and 25% filling densities, respectively.

**Figure 6:**
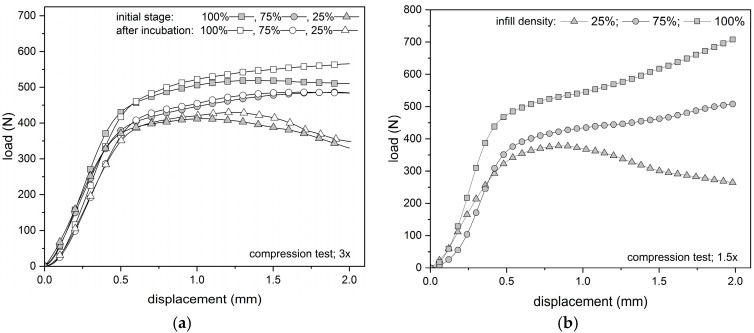
Comparison of the influence of infill density of PLA cylindrical specimens on the compressive characteristics: (**a**) average compressive curves before and post incubation (3× ratio samples), (**b**) average compressive curves of 1.5× ratio samples.

Analyzing the shapes of the average compression curves (Figure 6), linear and nonlinear regions can be seen. The test specimens were characterized by linear stiffness up to a force level of about 300 N and then deformed plastically. The infill density clearly affects the material’s response during compression, whereas the load exceeded the yield strength.

**Figure 7:**
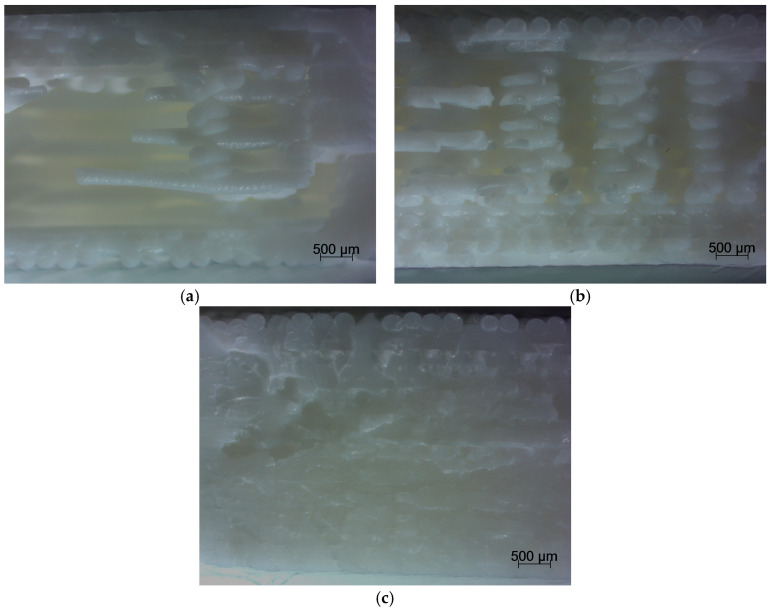
Comparison fracture of the specimen view: (**a**) fracture of the sample with an infill density of 25%, (**b**) fracture of the sample with an infill density of 75%, (**c**) fracture of the samples with an infill density 100%.

The character of fracture under tensile strain was brittle without delamination between the layers of printed samples (Figure 7). In contrast, during compression, regardless of the multiplicity of the specimens, the plastic nature of the failure was observed (the specimens assumed a barrel shape) with no signs of cracks between the layers. This is also confirmed by the shape of the compression curve.

By evaluating the results of the compression tests (Table 3), it can be noted that in the group of 1.5× specimens, the difference between values of compressive modulus was not statistically significant (*p* ≥ 0.05). Similarly, in the group of 3× specimens, the differences in modulus values were not statistically significant. The one-month incubation of the samples did not significantly affect the variation of the compressive modulus values. Reducing the filling density of the samples, both 1.5× and 3×, resulted in a reduction in the compressive strength values (statistically significant differences between the groups). The reduction in the UTS also depended on the height of the samples used in the tests. For 1.5× samples, the UTS varied from 28.42% (PLA_75) to 46,42% (PLA_25), and for 3× samples, it varied from 6.39% (PLA_75) to 17.90% (PLA_25) for a corresponding mass reduction of 7.22% and 14.45%, respectively. This showed the significant influence of the ratio of specimen height to diameter during the compression test on the results obtained. The effect of incubation on the compressive strength was statistically significant (*p* ≤ 0.05) only for specimens with 100% fill density.

**Figure 8:**
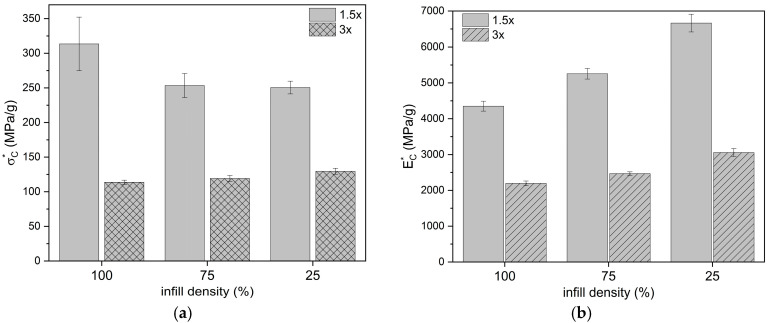

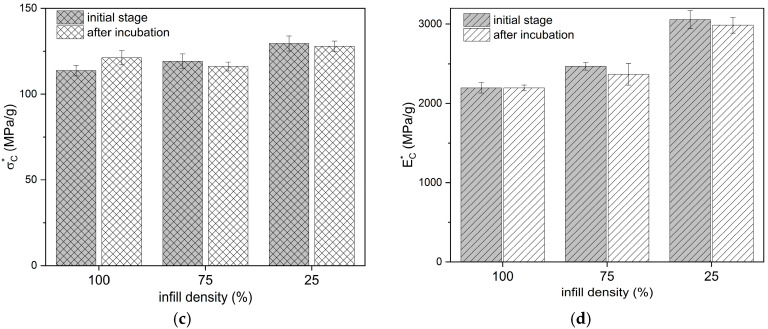
Comparison of the influence of infill density of PLA cylindrical specimens on the compressive properties in relation to mass: (**a**) compressive strength, (**b**) compressive modulus, (**c**) compressive strength of 1.5× ratio specimens, (**d**) compressive modulus before and post incubation (3× ratio samples).

Relating the value of Young’s modulus to the mass of the specimens tested showed that a reduction in the infill density did not reduce the compressive stiffness of the specimen (Figure 8a,c). For tensile strength, a decrease in tensile modulus was observed when the infill density was reduced from 100% to 75% (Figure 8b,d). A further reduction in infill density to 25% did not reduce the compressive strength in relation to mass.

### 3.2. Digital Image Correlation (DIC)

**Figure 9:**
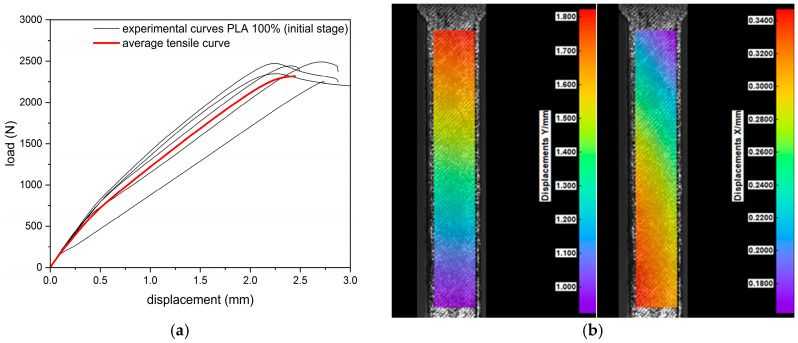
Results of tensile test: (**a**) tensile curves for specimens of 100% infill density, (**b**) digital image correlation displacement distribution maps in the longitudinal (Y) and transverse (X) directions.

**Figure 10:**
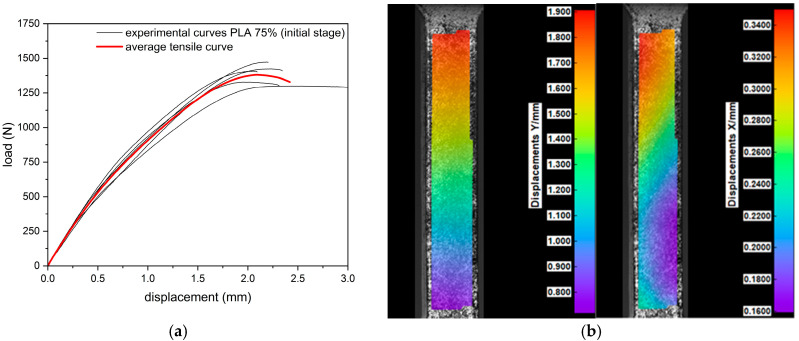
Results of tensile test: (**a**) tensile curves for specimens of 75% infill density, (**b**) digital image correlation displacement distribution maps in the longitudinal (Y) and transverse (X) directions.

**Figure 11:**
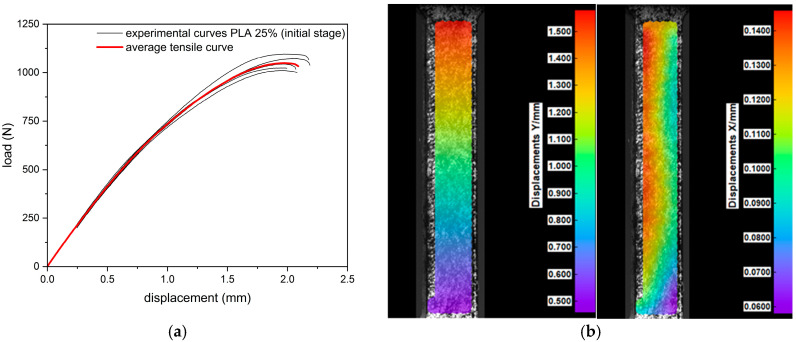
Results of tensile test: (**a**) tensile curves for specimens of 25% infill density, (**b**) digital image correlation displacement distribution maps in the longitudinal (Y) and transverse (X) directions.

**Figure 12:**
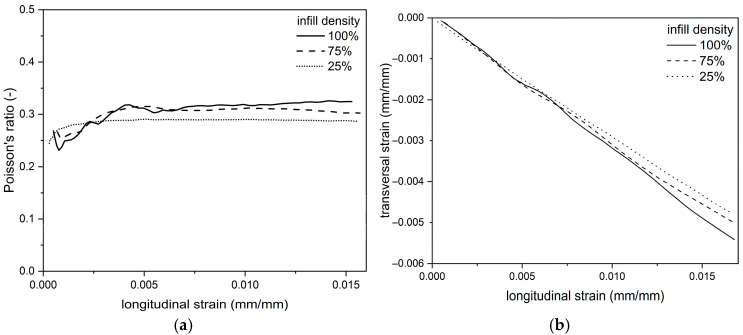
The curves obtained from DIC method for PLA sample materials with different infill densities: (**a**) Poisson’s ratio vs. longitudinal strain, (**b**) transverse strain vs. longitudinal strain.

Deformations in the longitudinal (Y) and transverse (X) directions were recorded by the digital image correlation (DIC) system. The results of the uniaxial tension tests coupled with their corresponding DIC tensile displacement contours at frames captured just before fracture are presented in Figure 9, Figure 10 and Figure 11. Based on the displacement maps, the strains for the two main directions were determined and the Poisson’s ratio values were calculated (Figure 12). Determination of the Poisson’s ratio using a digital image correlation system is possible through the use of a virtual gauge element. This analysis uses a virtual gauge element in the form of a polygon, within a predefined region of interest (ROI)—the area in which the Istra4D software V4.8.2.248 collects and averages the principal strains. Reducing the infill density of the PLA samples resulted in the same decrease in the Poisson’s ratio value for both infill densities, i.e., from 0.32 to 0.30 (Table 4).

### 3.3. Stress Relaxation

**Figure 13:**
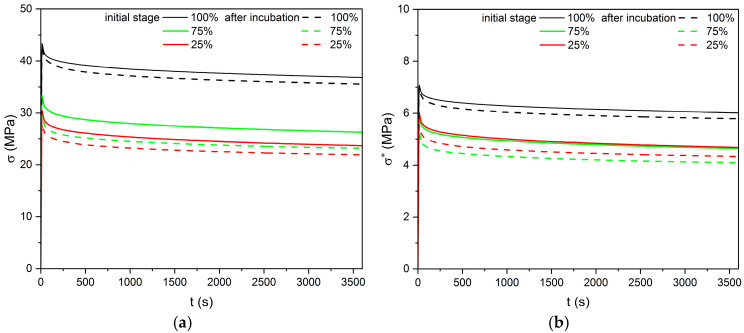
Stress-relaxation curves for PLA with different infill density: (**a**) stress relaxation vs. time, (**b**) stress relaxation related to mass vs. time.

The trend in stress relaxation was similar for all the specimens tested, with rapid relaxation in the first 100 s, followed by a slow slope to a stabilized stress value (Figure 13). Preliminary tests of stress relaxation at 2% strain highlighted differences between samples with 100% infill density and those with 75 and 25% infill (Table 5). As with tensile and compressive loading, a noticeable decrease in stress relative to the mass of the specimens is observed when the infill density is reduced from 100% to 75%, whereas further reductions in density do not result in a decrease in stress values.

## 4. Discussion

Reports on the influence of infill density on the mechanical properties of 3D-printed PLA are thoroughly documented by several authors [38,39,40]. The mechanical properties under tensile loading were of main interest in these publications. The result of this study concentrated on a more complex approach and provided data for tensile and compressive behavior, as well as stress relaxation under constant strain in PLA. Decreasing the infill density of 3D-printed PLA specimens resulted in a reduction in mass but also mechanical properties under tensile loading, including tensile strength and Young’s modulus, indicating deteriorated stiffness. Significantly, a comparison of these parameters to the weight of the specimens showed that a reduction in infill density from 75% to 25% did not significantly reduce them. In the case of compression specimens, only the strength decreased with a reduction of infill density, whereas the values of the elastic modulus did not change significantly between the test groups. While tensile testing data is fairly common in the literature for 3D-printed specimens, the current work presented here reveals full-field longitudinal and transverse displacement contour plots highlighting the different displacement patterns observed for the various infill densities. DIC was also used to measure Poisson’s ratio. The DIC method complements traditional testing approaches by offering a more comprehensive view of material behavior under stress.

Pandzic and Hodzic [41] indicated that the infill pattern has an influence on the tensile mechanical properties (tensile strength and elastic modulus), and they referred to values of ultimate tensile strength in the range from 38.40 to 45.57 MPa and Young’s modulus in the range from 2.10 to 2.4 GPa, for 60% infill density. In another work [38], the authors considered the density of the infill and its gradient, and also noted the dependence of the strength properties on these parameters. The compressive strength increased with the increase in the degree of filling. Considering the change in the printing density of cylindrical specimens in the range of 80% to 10%, they recorded compressive strength values ranging from 41.2 MPa to 0.8 MPa and modulus of elasticity values from 1500 MPa to 10 MPa, respectively. On the other hand, by isolating two printing zones (the outer one at a constant density level of 80% and the inner one varying from 60% to 20%), they recorded a decrease in compressive strength from 31.7 MPa to 10.7 MPa, and a decrease in modulus of elasticity values from 1200 MPa to 500 MPa, respectively [38]. Dave H. et al. compared the effects of three infill density levels of 80%, 70%, and 60% and three layer heights (0.1, 0.2, and 0.3 mm) on compressive strength, obtaining values in the range of 33.13–52.93 MPa, 34.85–43.62, and 26.46–33.54, respectively [39]. Hodzic D. and Pandzic A. [40] emphasized that the mechanical properties of 3D-printed PLA material depend heavily on the type of infill pattern. They compared different infill patterns—concentric, grid, gyroid octet, and triangle—as well as their densities (20%, 40%, 60%, and 80%). Their findings indicate that the grid pattern at an 80% infill density exhibited the highest compressive strength and modulus values. Specifically, for 20% infill, they recorded 29.1 MPa and 0.49 GPa; for 40%, 38 MPa and 0.54 GPa; for 60%, 44.6 MPa and 0.67 GPa; and for 80%, 54.1 MPa and 0.66 GPa. The tensile test described in the paper [42] examines 3D-printed PLA material with a infill density of 30%, comparing various infill patterns—Hilbert, gyroid, 3D honeycomb, stars, and honeycomb. The results indicate that the honeycomb infill exhibits the highest tensile strength, reaching 29.4 MPa.

The ratio of the printed part’s density to the bulk material’s density is directly proportional to the change in Poisson’s ratio, especially in orthotropic (directionally dependent) materials. Poisson’s ratio in 3D-printed materials is highly tunable and depends on a combination of material selection, microstructural design, print path, orientation, and density. Print direction and raster orientation can introduce anisotropy, making Poisson’s ratio vary depending on the direction of loading relative to the print layers [43]. The measured Poisson’s ratio value for 100% infill density of 0.32 is in good agreement with the data provided by Wang et al. [44] in the range of 0.32 to 0.33 depending on the raster orientation and reported in the paper [45] for printed PLA at 0.33. The slight differences in the variation of Poisson’s ratio values observed in our study, as well as in the literature, may be due to the dominant influence of the outer layers on the printed samples’ deformation in the longitudinal and transversal directions compared to the core deformation for the given density and infill pattern.

As the results from the literature analysis show, the infill density [4,38,39], type of infill pattern [41,42], and the direction of 3D processing [34,35,46,47] of the specimens influence their mechanical behavior. The processing (printing) direction leads to significant anisotropy in properties such as strength, stiffness, ductility, and failure modes. Specimens printed with layers aligned along the loading direction show much higher strength and stiffness compared to those printed upright or at higher angles (e.g., 90°) [48]. Tüfekci et al. showed that the printing direction has a major impact on the tensile strength. The obtained tensile strength values were at the level of 42, 36, and 25 MPa for PLA samples with 100% infill density and 0°, 45°, and 90° infill orientations, respectively [46], whereas the stress-relaxation results presented in [46] focused on the change in initial stress values from 16.33, 13.48, and 9.55 MPa, for the 0°, 45°, and 90° infill orientations to 13. 89, 11.81, and 8.21 MPa, after two hours of relaxation.

The stress relaxation of PLA material was the subject of the paper [47], in which the authors referred to Young’s modulus values of 3045 ± 3 MPa, 2914 ± 3 MPa, and 2932 ± 3 MPa for a 0°, 45°, and 90° infill orientation and normalized creep modulus values in the range of 0.85–0.89, 0.9–0.92, and 0.85–0.92 after about 10 min of relaxation for a 0°, 45°, and 90° infill orientation, respectively.

Systemic evaluation of how 3D-printed PLA performs under different load conditions can contribute to optimizing processing parameters, ensure material quality, and enable the reuse of PLA in new products, thus reducing waste and resource consumption. The results of this study can be used to evaluate how recycled and reprocessed PLA perform. Repeated PLA recycling can lead to decreases in tensile strength (from 20.58 MPa for virgin to 12.04 MPa for 10% virgin/90% recycled) and Young’s modulus (from 5.86 GPa for virgin to 4.74 GPa for 10% virgin/90% recycled) [49]. Some studies report that adding recycled PLA to virgin PLA can improve its mechanical properties, from 44.2 ± 2.18 MPa for virgin PLA to 52.61 ± 2.28 MPa for 25% virgin/75% recycled PLA [50].

Overall, increasing the infill density of 3D-printed PLA components enhances their mechanical properties, including tensile strength, Young’s modulus, fatigue resistance, and impact strength [51,52,53]. These improvements make high-density infill settings preferable for applications requiring robust and durable components. However, higher infill densities result in increased mass because more material is used to fill the internal structure of the printed object. The experimental data obtained in this study can be used in the design of 3D-printed components with consideration of different infilling densities in order to optimize the mass and mechanical properties of the printed components.

## 5. Conclusions

The closed-loop economy, which aims to improve sustainability, is turning the attention of manufacturers toward the thoughtful use of plastics throughout the value chain, forcing a different approach to product design and manufacturing. One such path may be to reduce the weight of the product. It may lower mechanical performance but can still provide environmental benefits by reducing the volume of plastics on the market and consequently plastic waste. The results obtained in this study show that reducing the mass of the samples by reducing the infill density from 100% to 75% reduces mechanical properties, but a further reduction to 25% does not significantly decrease the strength properties. When decreasing the infill density from 100% to 25%, a 40% mass reduction was observed, which resulted in a 56% decrease in tensile strength and a 53% decrease in Young’s modulus. A 46% and 20% decrease in compressive strength was also observed, depending on the sample multiplication factor (1.5× and 3×, respectively). This shows that adjusting the infill density and appropriate design strategy for 3D-printed PLA can minimize material use while maintaining mechanical strength. This provides sustainability benefits by reducing material consumption, energy use, and production time. Using less material aligns with circular economy principles and supports the use of recycled or biodegradable feedstocks, further reducing environmental impact. PLA is biodegradable and, when sourced sustainably, has a lower carbon footprint than petroleum-based plastics. Incorporating recycled PLA in future studies can further reduce environmental impact. Increasing the infill density of 3D-printed PLA parts results in a heavier object with enhanced mechanical properties, including tensile strength, stiffness, and fatigue resistance. However, the optimal infill density may vary depending on the specific application requirements, balancing material use and the desired mechanical performance. In addition, the ability of PLA to slowly biodegrade is an advantage in favor of its use as a substitute for many stable polymers, the use of which is temporarily defined or limited. Lightweight components manufactured using 3D-printing technology are used as non-load-bearing structures in many areas of engineering, e.g., as housings and covers, prototypes, and conceptual and educational models, as well as everyday or decorative elements.

However, the methods of assessing the material parameters sought by the manufacturer should always be analyzed. In this work, attention was paid to the influence of both the types of tests and the shapes of the samples used in the tests, as well as the level of hydration and mass reduction, on the mechanical properties of PLA samples obtained by 3D printing. The data can be used for numerical analyses of objects manufactured using 3D printing with the application of various parameters, which can speed up the product optimization process. Using fused deposition modeling can support the transition towards a circular economy in the construction industry, particularly in medicine, which requires product personalization. The study has several limitations such as focusing on a limited set of printing parameters, limited time of water absorption, and lack of thermal and photo aging. The search for the load transfer capacity of 3D-printed PLA should be completed by testing under dynamic load. Future work on assessing the effect of mass reduction will focus on obtaining an optimal set of 3D-printing parameters to increase the ability to carry static and dynamic loads. Mechanical properties’ evaluation will be enhanced by analysis of how the infill density, infill pattern, and raster orientation affect stress concentration, fracture resistance, and the overall mechanical performance of 3D-printed parts.

## Figures and Tables

**Table 1:** Basic printing parameters.

Printing Parameters	Value
speed of monolithic layers, mm/s	35
speed of non-monolithic layers, mm/s	50
working table temperature, °C	50
speed of extruder travel over areas where material was not applied, mm/s	80
thickness of the invisible layers of the upper part of the sample, mm	0.36
thickness of the outer top layer, mm	0.72
filament withdrawal distance, mm	5
printing temperature (print head), °C	220
infill type	Diamond Fill Fast
layer height, mm	0.2
number of contours	2
raster angle, °	0/90
density of infill, %	100, 75, 25

**Table 2:** Mechanical properties obtained in tensile test.

	UTS [MPa]	E [MPa]
PLA_100	60.04 ± 2.24 ^a,b^	2645.05 ± 204.15 ^a^
PLA_100i	55.50 ± 1.16 ^b^	2693.63 ± 123.01
PLA_75	34.66 ± 1.59 ^a^	1635.93 ± 149.27 ^a^
PLA_75i	33.17 ± 1.20	1756.61 ± 87.87
PLA_25	26.24 ± 0.77 ^a^	1245.41 ± 83.79 ^a^
PLA_25i	25.98 ± 0.47	1329.15 ± 97.23

Note: PLA_100/PLA_75/PLA_25–100%/75%/25% of infill density, i—specimens after hydration. Note: The same lowercase letters within the columns indicate a statistically significant difference between the groups (*p* ≤ 0.05).

**Table 3:** Mechanical properties obtained in compression tests.

	σ_c_ [MPa]	E_c_ [MPa]
1.5_PLA_100	36.24 ± 3.32 ^a,d^	504.60 ± 9.20
1.5_PLA_75	25.94 ± 1.82 ^a^	513.60 ± 11.91
1.5_PLA_25	19.42 ± 0.44 ^a,e^	516.60 ± 7.12
3_PLA_100	26.59 ± 0.80 ^b,c,d^	513.2 ± 9.68
3_PLA_100i	28.92 ± 0.96 ^c^	513.2 ± 4.87
3_PLA_75	24.89 ± 0.88 ^b^	515.00 ± 7.01
3_PLA_75i	25.32 ± 1.62	515.00 ± 8.74
3_PLA_25	21.83 ± 1.01 ^b,e^	514.60 ± 3.61
3_PLA_25i	22.22 ± 1.24	517.40 ± 3.14

Note: PLA_100/PLA_75/PLA_25–100%/75%/25% of infill density, i—specimens after hydration, 1.5/3—height to diameter ratio. Note: The same lowercase letters within the columns indicate a statistically significant difference between the groups (*p* ≤ 0.05).

**Table 4:** Comparison of Poisson’s ratio for PLA samples determined by DIC.

	Poisson’s Ratio [-]
PLA_100	0.32 ± 0.01 ^a,b^
PLA_75	0.30 ± 0.01 ^a^
PLA_25	0.30 ± 0.01 ^b^

Note: PLA_100/PLA_75/PLA_25–100%/75%/25% of infill density. Note: The same lowercase letters within the columns indicate a statistically significant difference between the groups (*p* ≤ 0.05).

**Table 5:** Values of maximal $\sigma_{max}$, residual $\sigma_{\infty }$, and normalized $\sigma_{normalized}$ stress relaxation for PLA samples with different infill densities.

	$\sigma_{max}$ [MPa]	$\sigma_{\infty }$ [MPa]	$\sigma_{max}^{*}$ [MPa/g]	$\sigma_{\infty }^{*}$ [MPa/g]	$\sigma_{normalized}$ [-]
PLA_100	43.53 ± 1.54 ^a,b^	36.80 ± 3.33 ^a,b^	7.11 ± 0.25	6.02 ± 0.28	0.85 ± 0.01
PLA_100i	43.16 ± 0.40	35.55 ± 0.29	7.03 ± 0.03	5.79 ± 0.02	0.82 ± 0.00
PLA_75	33.60 ± 1.31 ^a^	26.26 ± 1.10 ^a^	5.93 ± 0.23	4.64 ± 0.20	0.78 ± 0.00
PLA_75i	30.09 ± 1.16	23.20 ± 1.32	5.32 ± 0.21	4.10 ± 0.24	0.78 ± 0.08
PLA_25	30.62 ± 0.76 ^b^	23.72 ± 1.02 ^b^	6.04 ± 0.15	4.83 ± 0.05	0.78 ± 0.02
PLA_25i	28.11 ± 0.90	21.94 ± 0.04	5.55 ± 0.18	4.33 ± 0.01	0.79 ± 0.03

Note: PLA_100/PLA_75/PLA_25–100%/75%/25% of infill density. i—specimens after hydration. Note: Same lowercase letters within the columns indicate statistically significant difference between the groups (*p* ≤ 0.05).

## Data Availability

The original contributions presented in this study are included in the article. Further inquiries can be directed to the corresponding author.
